# Microbial interactions of EDTA: recent advances and biological applications in the context of natural product modulation

**DOI:** 10.3389/frabi.2026.1767602

**Published:** 2026-04-23

**Authors:** Gunanidhi Sahoo, Aditi Jena, Sudipta Kumar Patra, Sujogya Kumar Panda, Satyanarayan Pal

**Affiliations:** 1 Department of Zoology, Utkal University, Bhubaneswar, Odisha, India; 2 Department of Orthopedics, Kalinga Institute of Medical Sciences, Bhubaneswar, Odisha, India; 3 Centre for Biotechnology, Siksha ‘O’ Anusandhan (Deemed to be University), Bhubaneswar, Odisha, India; 4 Department of Chemistry, Utkal University, Bhubaneswar, Odisha, India

**Keywords:** antimicrobial property, biofilm, EDTA, natural therapeutics, synergistic effect

## Abstract

**Background and objectives:**

Ethylenediaminetetraacetic acid (EDTA) and its salts have been the choice of chelating agents since the 1940s. This review presents an updated details of their synthesis, general biology, ecotoxicological aspects, and applications as antimicrobial and antibacterial agents in combination with natural products.

**Methods:**

Relevant research papers were retrieved from PubMed, Web of Science, and Google Scholar through November 2025. Experimental uses of EDTA were excluded. The search terms used were “EDTA” AND “ECOTOXICOLOGY”; “EDTA” AND “GENOTOXICITY”; “EDTA” AND “ANTIMICROBIAL ACTIVITY”; “EDTA” AND “ANTIBACTERIAL ACTIVITY” AND “DENTAL” and “EDTA” AND “ANTICANCER ACTIVITY”, “EDTA IN COMBINATION WITH NATURAL PRODUCTS”.

**Results:**

Sodium/calcium salts of EDTA are water soluble, and their antiseptic efficacy is pH-dependent. They are effective against both Gram-positive and Gram-negative bacteria, as well as pathogenic yeasts, and adversely affect bacterial cell walls, thereby destabilizing biofilms. Multiple nature-derived compounds and standard antibiotics, in combination with EDTA and other therapeutic agents, minimize biofilms in intravascular and urinary catheters. It chelates various metal ions (including heavy metals) into a redox-inactive state, and thereby reduce their toxicity. Furthermore, it was shown to enhance the antimicrobial and antibacterial efficacy of various natural therapeutics when used together.

**Interpretation and conclusions:**

EDTA is a stable, readily available, affordable and comparatively safer chelating agent with antibacterial, antifungal, and antibiofilm properties. EDTA is now found to produce a synergistic effect when combined with natural therapeutics on their antimicrobial/antibiofilm activities. This approach proved fruitful in enhancing the capabilities of natural antibiotics against multidrug-resistant bacteria and in reducing the toxic effects of EDTA.

## 1 Introduction

Chelating agents are of utmost importance in chemical and biological sciences. Innumerable chelating agents are designed and synthesized as required in a range of different chemical and medical frontline areas. These chelating agents possess high metal-binding affinity and form metal-chelate complexes with high thermodynamic stability due to the chelating effect of the coordinating ligands. A chelated metal ion will thus move to a new chemical environment and display modified sets of chemical activities, suppressing the chemistry of bare metal ions. Commonly used chelators in the field of biology include deferoxamine, deferiprone, deferasirox, EDTA etc. Deferasirox (DFX), and EDTA, along with its salts (Flora and Pachauri, 2010). Among the available chelators, EDTA is the most widely used, as it finds applications not only in biology but also in chemical sciences and various industrial sectors. The very high metal affinity of EDTA has led to its exploration in the treatment of heavy metal poisoning in the human body through chelation therapy (Oviedo and Rodríguez, 2003; Klaassen, 2006; Hoffman, 2014; Sultan et al., 2017). It was first synthesized and patented in Germany in 1935 by F. Munz (Paolieri, 2017; Janusz Ryczkowski, 2019) with its initial application in the dye industry in the 1940s as a means of binding and extracting calcium ions (Hoffman, 2014; Sultan et al., 2017). The calcium-binding ability was also noticed by Martin Rubin of Georgetown University Medical Centre in Washington, USA (Hoffman, 2014; Sultan et al., 2017). He promoted the use of EDTA as a chelating agent in the treatment of lead poisoning. The chelation therapy for lead poisoning using EDTA was started in 1950 (Oviedo and Rodríguez, 2003; Sultan et al., 2017). The chelated lead got excreted from the body through urine without being accumulated inside the body. It is the prescribed chelator for treating lead and other heavy metal poisonings to date. Its potential use in treating atherosclerotic heart disease was discovered by Mosher (Clarke et al., 1956) with encouraging results in decalcifying the diseased coronary arteries in patients with severe angina (Kumric et al., 2023).

Miriam et al. reported appreciable treatment effects on bone defects introduced into rat jaws using a mixture of 10% citric acid, 17% EDTA, and 1.23% sodium lauryl sulphate (Scelza et al., 2003). It was also noted that intravenously administered CaNa_2_EDTA improves kidney problems, loss of appetite, abdominal pain, and other related symptoms.

It was found to be safe in small amounts when used as a preservative in various food items. Even the safer use of EDTA in eye drops has also been reported (Cristaldi et al., 2018). The high level of EDTA in the body usually led to extremely low levels of calcium and severe damage to the kidneys. The toxic effects of EDTA can include diarrhoea, nausea, headache, vomiting, and fever (Flora and Pachauri, 2010). This review highlights the chemistry and various biomedical applications of EDTA, as well as its toxic effects, in a focused manner.

## 2 Literature search strategy

To ensure transparency and reproducibility, relevant research papers were retrieved from PubMed, Web of Science, and Google Scholar through November 2025. Experimental uses of EDTA were excluded. The search terms used were “EDTA” AND “ECOTOXICOLOGY” (PUBMED 41, WOS 09), “EDTA” AND “GENOTOXICITY” (PUBMED 531, WOS 65), “EDTA” AND “ANTIMICROBIAL ACTIVITY” (PUBMED 167, WOS 337), “EDTA” AND “ANTIMICROBIAL ACTIVITY” AND “DENTAL” (PUBMED 31, WOS 00), and “EDTA” AND “ANTICANCER ACTIVITY” (PUBMED 14, WOS 59). Studies discussing the biological, ecological, or medical uses of EDTA were included; those focusing solely on synthetic chemistry or experimentation were excluded. To aid in comparative analysis, articles were categorized thematically into the following areas: ecotoxicology, genotoxicity, antimicrobial/antibiofilm activity, synergistic effects with natural compounds, and therapeutic uses. This methodical approach makes it easier for readers to understand how the review’s papers were selected, organized, and summarized.

## 3 Synthesis, structure, and chemical nature of EDTA

**Figure 1:**
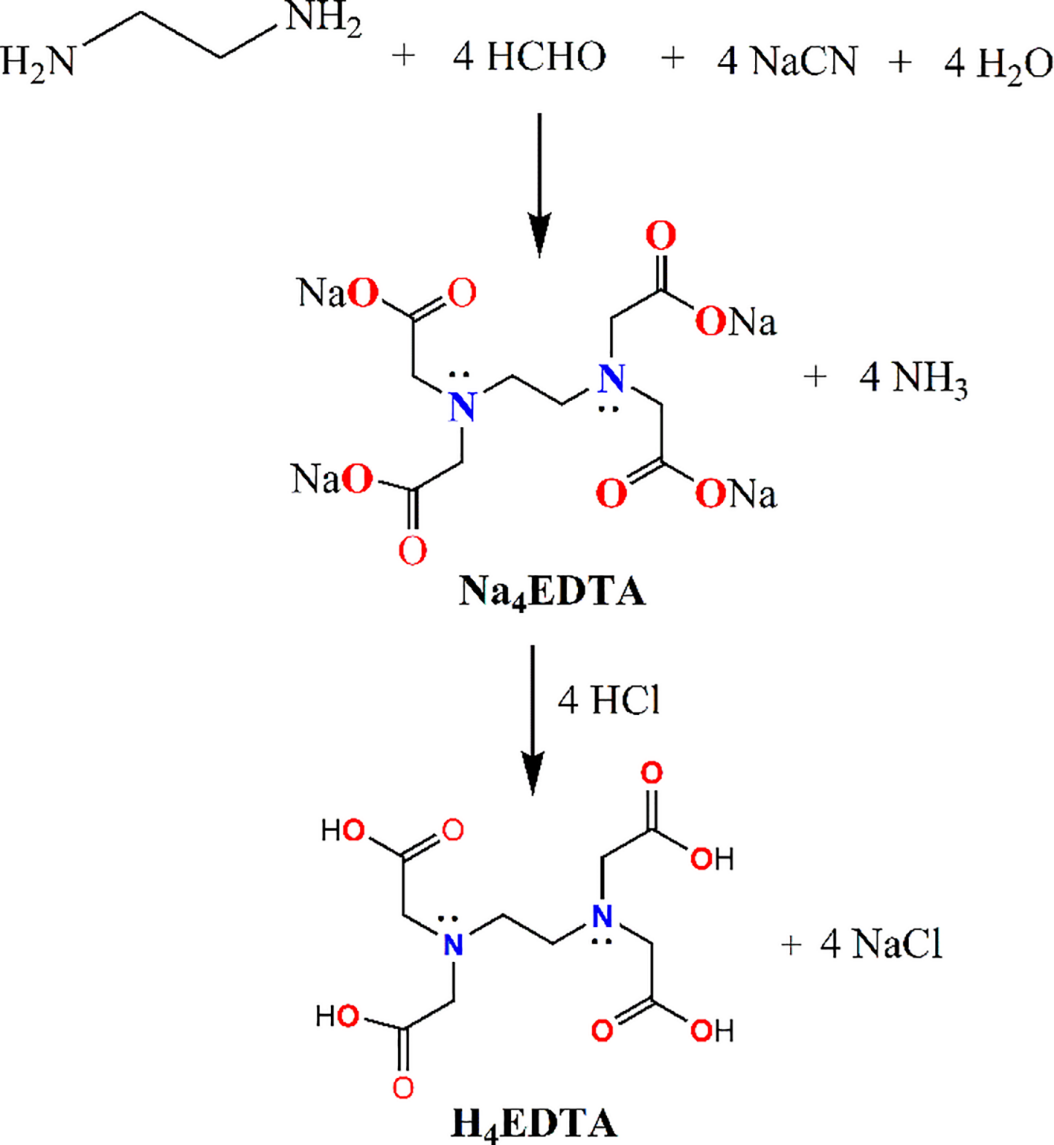
Synthesis and structure of H_4_EDTA.

The first synthesis of EDTA was reported by F. Münz in 1935 (Paolieri, 2017; Ryczkowski, 2019), who successfully prepared it from the reaction of ethylene diamine and chloroacetic acid. The industrial-scale production at present is achieved through the reaction of ethylenediamine, formaldehyde, and NaCN (**Figure 1**) (Ryczkowski, 2019). The reaction yielded the Na-salt of EDTA, which, upon acidification, was converted to the acidic form through protonation of carboxylate groups to edetic acid (H_4_EDTA).

The abbreviation EDTA is used for the original compound and sometimes also for its disodium salts. EDTA is an acidic, white, odorless, and crystalline solid that is soluble in water and aqueous alkaline medium and found to be highly stable for an extended period of time. On the contrary, EDTA and its salts are insoluble in alcohols, ethers, and other commonly used organic solvents. It is anionic and forms a tetra-negative anion that acts as a potent chelator for alkali, alkaline earth, and transition metal ions. The solubility of H_4_EDTA in metal hydroxide solution is due to the formation of water-soluble salts (EDTA^4-^) or metal-EDTA chelates.

**Figure 2:**
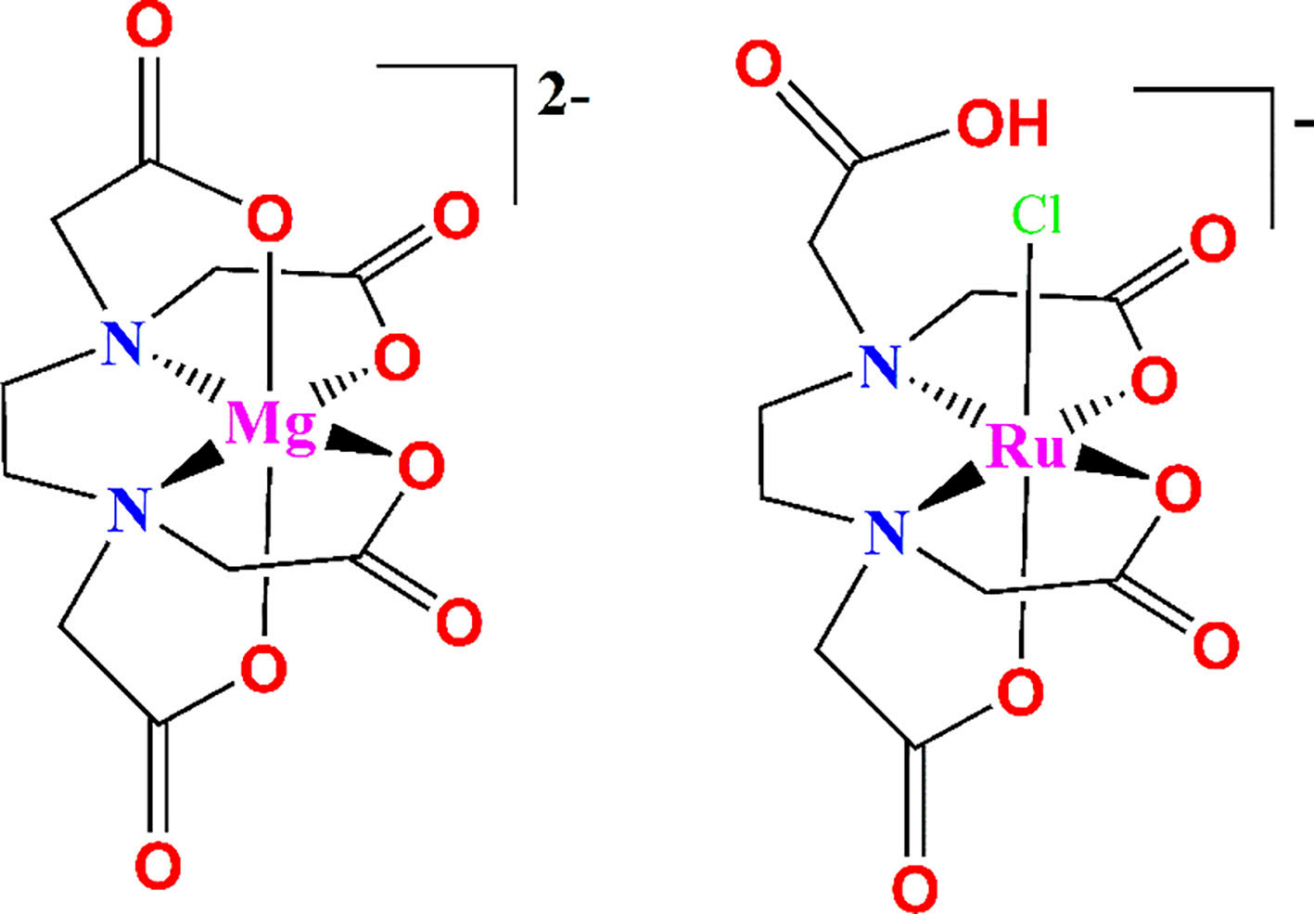
Two different coordination modes of EDTA.

The polyprotic edetic acid exerts its chelation to the metal ions through four carboxylate-O and two amine -N atoms as a hexadentate ligand. Other coordination modes are also found when not all the -COO^-^ groups are part of the chelation. Hence, it can adopt various coordination modes with interacting metal ions (**Figure 2**) (Jolley et al., 1998; Mudsainiyan and Chawla, 2015).

**Figure 3:**
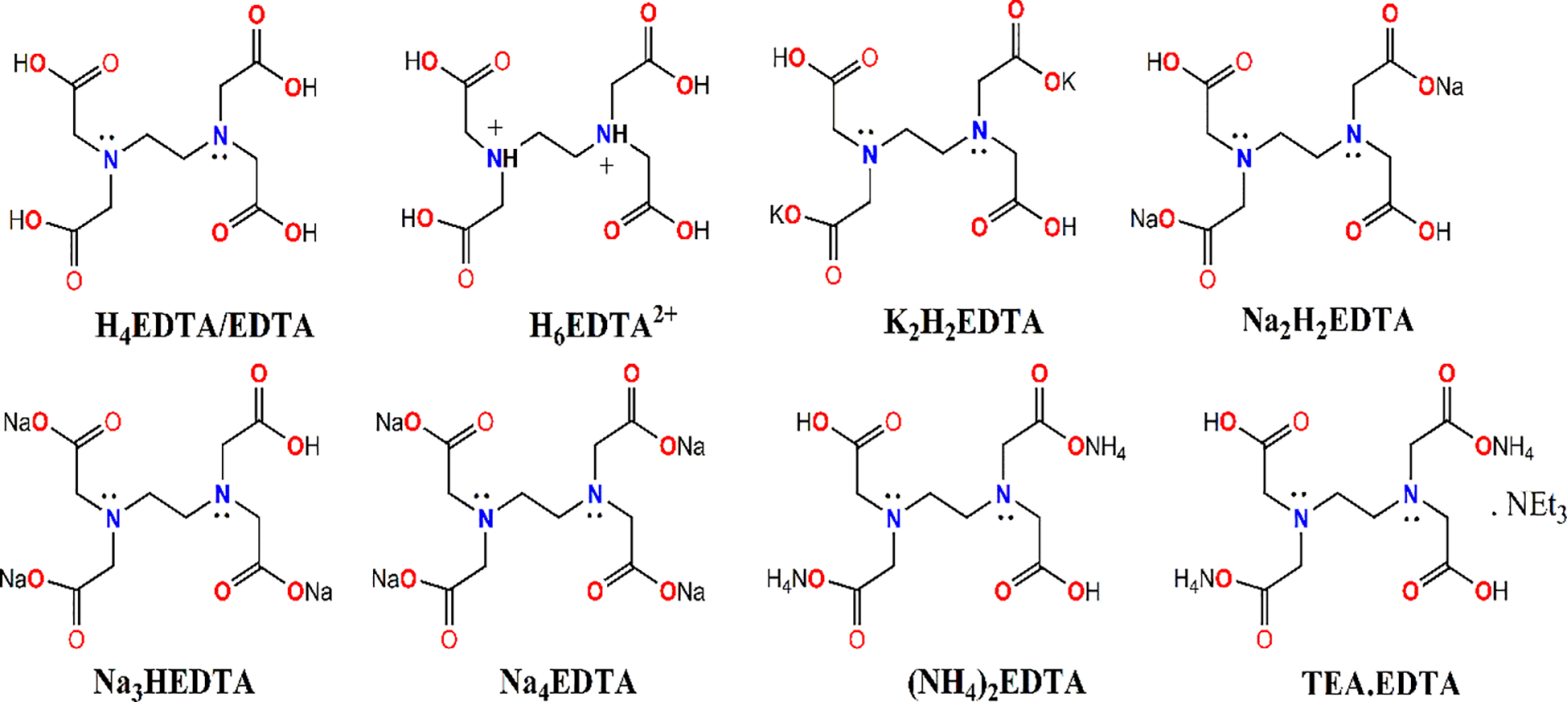
EDTA and its different salts.

It is to be noted that only the deprotonated carboxylate-O atoms affect the binding of metal ions. Thus, the metal chelation was found to proceed at basic pH. At acidic pH, the amine N’s get protonated and a fully protonated [H_6_EDTA]^2+^ form exists, whereas the fully deprotonated form [EDTA]^4-^ dominates at higher pH (Banfi et al., 2007; Finnegan and Percival, 2015). The different salts of EDTA used for various purposes are depicted in **Figure 3**. It can form water-soluble, highly stable metal complexes with most multivalent metal ions, including Na^+^, Ca^2+^, Fe^2+^, Co^2+^, Zn^2+^, Cu^2+^, and Pb^2+^, as well as Fe^3+^. This high stability of the complexes arises from the metal ions achieving a saturated coordination sphere and being surrounded by a cage-like structure. The metal ion, thus surrounded by EDTA, acquires a new chemical identity compared to its observed behavior in the free ionic state.

The stability of EDTA-metal complexes with different metal ions is found to be in the order of Na^+^< Ca^2+^< Fe^2+^< Co^2+^< Zn^2+^< Cu^2+^< Pb^2+^< Fe^3+^ (Lanigan and Yamarik, 2002). The complexation results in the shifting of cationic metal ions to an electron-rich coordination sphere, and the formation of anionic metal complexes; hence, an altered redox potential is observed for the corresponding metal ions, which ultimately enhances their separation from the aqueous phase.

## 4 General biology of EDTA

EDTA, when administered intravenously, distributes throughout the body via the bloodstream and binds to various metal ions as a metal-EDTA complex, which is then excreted through the kidneys and liver (Sultan et al., 2017). The movement of [^51^Cr]-EDTA, a radioactive tracer, was studied in a dog model, where the metal chelate was found to move passively across the epithelium of the gastrointestinal tract (McKay et al., 1994). The [^51^Cr]-EDTA was rapidly moved in the ileum, followed by the colon and stomach, without any detectable build-up in the dog’s body. The mechanism of [^51^Cr]-EDTA movement is thought to be via a shunt pathway from the gut lumen (McKay et al., 1994).

When Ca-EDTA was administered to rats, the compound was rapidly absorbed and cleared from the body within one hour. Only a tiny portion of the substance (<0.1%) was found to be oxidized and expired as CO_2_. EDTA was not found to accumulate in any of the organs, and most of the compound (95-98%) was excreted from the body in the form of urine (Foreman et al., 1953).

Further studies on rat models also revealed high excretion of disodium EDTA through feces and urine. When rats were administered varying amounts of disodium EDTA for 12 weeks, up to 82.2% was excreted from the body. The feces contained approximately 98% of the excreted disodium EDTA, with the remaining amount being found in urine (Lanigan and Yamarik, 2002).

The CaNa_2_EDTA is flushed out of the human body unchanged by the kidneys through both glomerular filtration and tubular excretion. It was found that approximately 5% of the administered CaNa_2_EDTA is absorbed in the human body through the GI tract. Further, it did not show any radioactivity in the bloodstream after administration (Foreman et al., 1953; Lanigan and Yamarik, 2002). Oral administration of calcium EDTA and sodium EDTA shows poor GI tract absorption in humans but increases the amount of calcium in the stool (Spencer, 2006).

## 5 Biological and medical applications of EDTA

### 5.1 Antiseptic properties of EDTA

EDTA is used as a component in the manufacture of antiseptics (Ntzimani et al., 2011). Some reports suggest that its efficacy depends on the pH of the treatment environment. Moreover, several readily available formulations contain either EDTA or its salts, e.g., sodium salts (disodium, trisodium, tetrasodium salts; cupric disodium, magnesium disodium, ferric sodium), ammonium salts (ammonium, di-ammonium), and potassium salts (dipotassium which are known to be antibacterial and act as antiseptics (Sherertz et al., 2006; Finnegan and Percival, 2015). Since pH plays a significant role in determining the potency of these compounds, the British Pharmacopoeia specifies the composition with precise pH (5% disodium EDTA, pH=4.0 to 5.5; trisodium EDTA, pH=7.0 to 8.0; tEDTA; pH=8.5 to 10.0).

### 5.2 Antimicrobial properties of EDTA

The antibacterial effect of EDTA has been known for over 50 years, as it has been shown to be broad-spectrum, effective against both Gram-positive and Gram-negative bacteria, including pathogenic yeasts (Brown and Richards, 1965; Finnegan and Percival, 2015; Pinheiro et al., 2021; Deilamani et al., 2023). The effect of EDTA varies from species to species in yeasts through the formation of complexes with membrane-bound Mg^2+^and Ca^2+^ ions (Finnegan and Percival, 2015). Yeasts produce siderophores to accumulate iron to maintain their life cycles. EDTA binds to iron, thereby strongly inhibits siderophore formation and the subsequent growth of fungi (Amich and Calera, 2014; Finnegan and Percival, 2015).

Due to its ability to chelate and adversely affect the cell walls of bacteria, as well as destabilize biofilms by removing calcium, magnesium, zinc, and iron ions, EDTA and its components are highly effective in eliminating existing biofilms and preventing biofilm formation, colonization, and proliferation. EPS, a heterogeneous matrix of polymers that includes polysaccharides, proteins, nucleic acids, glycoproteins, metal ions, and phospholipids, can sequester and degrade therapeutic agents, potentially causing a delay in the healing process of many chronic wounds. Disrupting this protective biofilm EPS in wounds facilitates healing by allowing antimicrobials to penetrate and kill the EPS-protected bacteria. EDTA decreases the cation concentration, reduces the crosslinking of LPS molecules, and thereby increases the solubility of EPS in water, making it more available to antimicrobial agents and facilitating its extraction from a variety of bacteria (Banin et al., 2006; Juda et al., 2008; Finnegan and Percival, 2015).

#### 5.2.1 Antibiofilm properties of EDTA

Chen and Stewart (Chen and Stewart, 2002) evidenced for the first time that EDTA can eradicate mixed biofilms. The mechanism involved electrostatic interactions, which contribute to biofilm cohesion, and iron cations are potent cross-linkers of the biofilm matrix, facilitating the dispersal of biofilm bacteria. Banin et al. (2006) observed an antibiofilm effect of EDTA, evidenced by the killing of *P. aeruginosa* cells (CLSM confirmed by mushroom-like structures). The authors also explained not only the killing of the cells but also the causes of biofilm dispersal (>99%). The authors also reported 100% effectiveness when EDTA (50 mM) was combined with gentamicin (50 μg/mL) in Tris buffer. Almeida et al (De Almeida et al., 2016). studied the efficacy of EDTA on 48-hour-old *Enterococcus faecalis* biofilms. The authors employed advanced techniques, including quantitative polymerase chain reaction (qPCR), to detect 16S RNA genes, in addition to the traditional method of counting and colony-forming units (CFU). Around 99% detachment was observed from the coverslips or biofilms using 17% EDTA. The CFU was lower than that of the control, but the efficacy was also lower (12%). qPCR data revealed 7-8% damage after EDTA treatment. *E*. *coli*, among others, is the primary causative agent of urinary tract infections and biofilm production in Foley catheters. Ayyash et al. (2019) established a novel non-antibiotic combination of triclosan and EDTA (10 mg ml^-1^/30 mg ml^-1^) to significantly eradicate and prevent biofilm formation of the tested *E. coli* strains on such catheters.

### 5.3 Synergistic effect of natural antibiotics and EDTA as antibiofilm and antimicrobial agents

**Figure 4:**
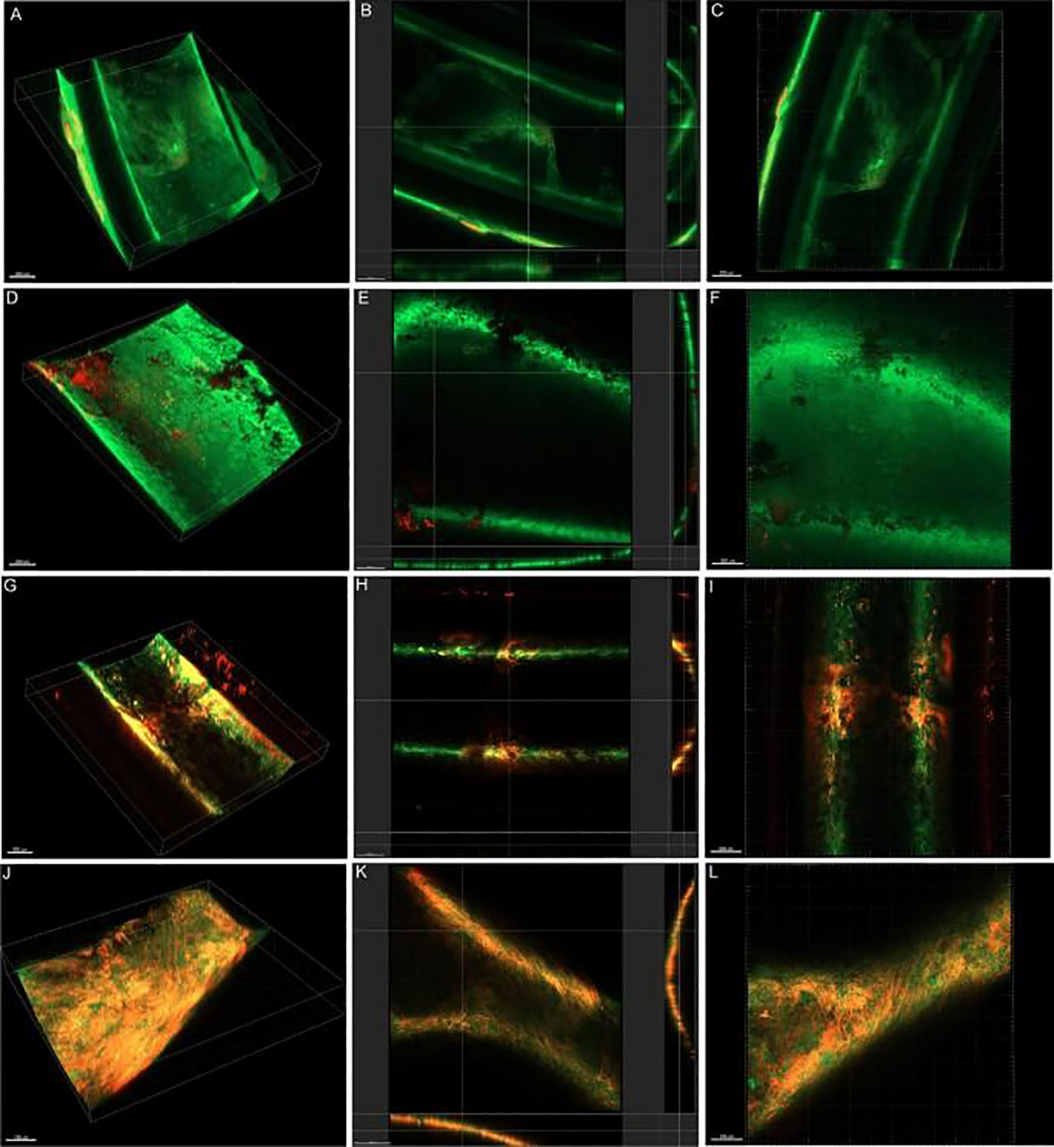
Confocal imaging analysis (3D and cross-sectional). **(A–C)** PBS-treated, **(D–F)** Colistin-treated, **(G–I)** EDTA-treated, **(J–L)** Colistin-EDTA combination-treated catheter-related biofilm infection of colistin-resistant *K. pneumoniae in vivo* (adopted from (Shein et al., 2021).

Nisin is a known natural antimicrobial agent against Gram-negative bacteria. Yüksel et al. (2018) observed synergy in the biofilm formed by *Salmonella typhimurium* when EDTA was combined with nisin. However, when the same combination was tested in a mixed biofilm model (11 bacteria), no such activity was observed compared with EDTA alone. Recently, Shein et al. (2021) established, for the first time, the combination of colistin and EDTA as a method for eradicating *K. pneumoniae* biofilms (*in vitro* and *in vivo*). The constituents (1 µg/mL colistin + 12 mg/mL EDTA) are promising for reducing and potentially even eradicating biofilm with an initial exposure of 6 hours (*in vitro*, crystal violet assay). Later, an *in vivo* catheter-infected experiment using the C57BL/6 mouse model confirmed a reduction in viable biofilm cells in treated animals infected with colistin-resistant *K. pneumoniae.* Confocal imaging (**Figure 4**) revealed a progressive decrease in bacterial population in several internal organs, as well as the tissue surrounding the catheter, in treated mice. The expression of several virulence genes is altered in response to stress in the host, for example, the gene mrkD (responsible for biofilm adhesion and the gene luxS (involved in quorum-sensing regulation) (Shein et al., 2021).

In an effort to improve the effectiveness of phytotherapeutics, Hamoud et al. studied two- and three-drug combinations, including thymol, EDTA, and vancomycin (Hamoud et al., 2014). The thymol-EDTA and thymol-EDTA-vancomycin combination proved to be more effective against *Staphylococcus aureus* (MRSA NCTC 10442) and *Escherichia coli* than the individual effect of the three drugs. By rupturing bacterial membranes, thymol, derived from thyme, effectively combats multidrug-resistant (MDR) bacteria. By weakening Gram-negative bacteria’s outer membrane and making them more vulnerable to antibiotics, EDTA (Ca²^+^ and Mg²^+^) amplifies this action. EDTA breaks down the barrier that prevents typically large antibiotics like vancomycin from reaching their targets by disrupting the structural integrity of lipopolysaccharides. This method shows how phytochemicals and traditional antibiotics can work in concert to reduce antimicrobial resistance (AMR).The discovery that current antibiotics can be “repurposed” or “resensitized” against resistant organisms when combined with natural and chelating chemicals is highly pertinent to the treatment of antimicrobial resistance (AMR). The study also showed a significant increase in potency against Gram-positive *S. aureus*, comparable to that against Gram-negative *E. coli*. This demonstrates that the mechanism underlying the synergy is multifactorial, with vancomycin gaining access to its peptidoglycan target after EDTA impairs the outer membrane and thymol further disturbs membrane lipids. A logical combination strategy is demonstrated by this progressive deterioration of bacterial defenses, which involves employing natural substances to overcome physical barriers and reestablish antibiotic access.

**Figure 5:**
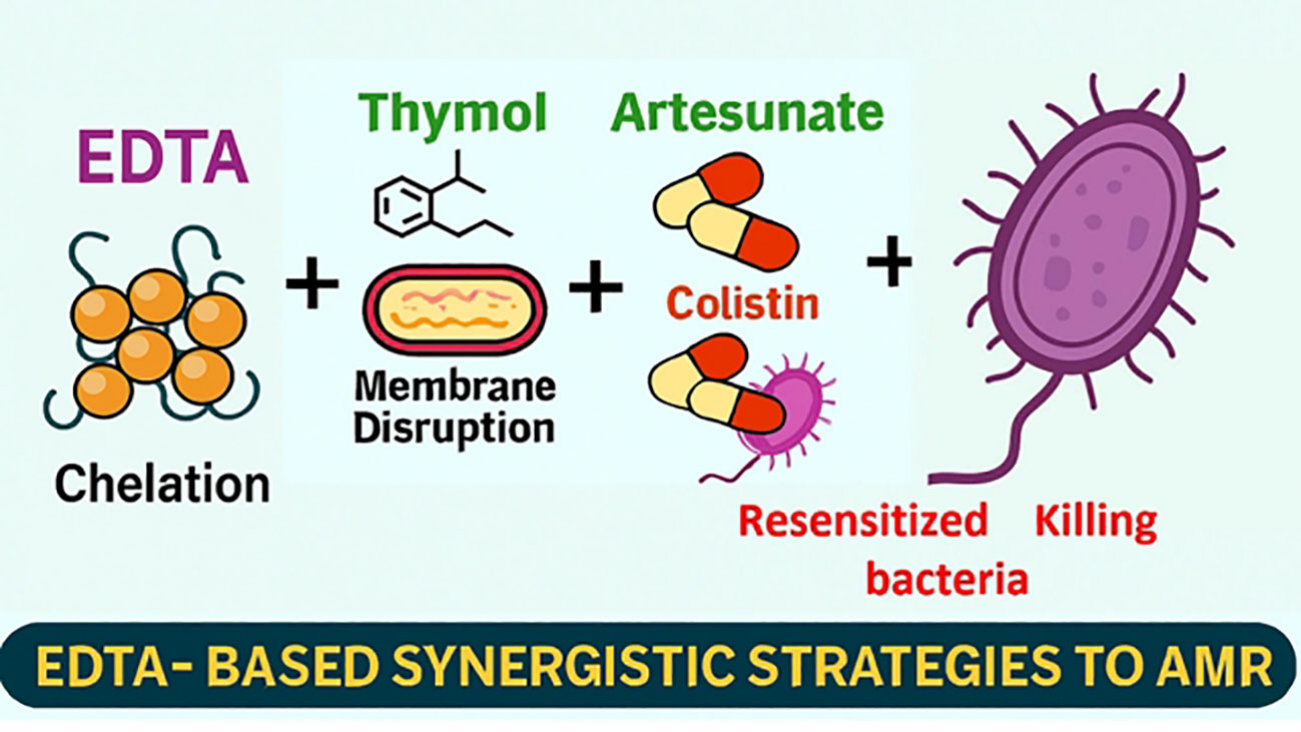
Thymol, artesunate, EDTA, and colistin work synergistically against colistin-resistant and MDR bacteria.

A similar study (Zhai et al., 2025) reveals that AEC, a combination of artesunate (AS), EDTA, and colistin (COL), exhibits vigorous synergistic antibacterial activity against both mcr-1-negative and mcr-1-positive MDR *Salmonella* strains. Individually, As and EDTA showed little antibacterial impact, but together with collistin, they significantly enhanced its effectiveness, reducing colistin’s minimum inhibitory concentrations (MICs) by up to 60,000-fold. This was confirmed in both *in vitro* and *in vivo* models, where AEC-treated mice had lower bacterial levels in organs. The synergy is attributed to EDTA destabilizing the bacterial membrane, while As increases membrane permeability and inhibits efflux pumps, facilitating COL’s action. Additionally, the combination disrupts the proton motive force and generates reactive oxygen species, which are crucial for bacterial metabolism. Transcriptomic and metabolomic analyses revealed down regulation of virulence pathways and accumulation of toxic metabolites. Overall, the study suggests a robust multi-targeted mechanism that enhances colistin’s effectiveness, potentially paving the way for repurposing artesunate as an antibiotic adjuvant against antimicrobial resistance (**Figure 5**).

According to a study Vale et al. (2019), EDTA greatly increases the antibacterial properties of some phytochemicals, such as cuminaldehyde and indole-3-carbinol, against both planktonic and biofilm forms of *E. coli* and *Staphylococcus epidermidis*. Even at doses close to the minimum bactericidal concentration, these phytochemicals have high synergistic activity when combined with EDTA, resulting in approximately 100% inactivation of single and dual-species biofilms, despite their poor antibacterial activities on their own. The effectiveness of EDTA is derived from its capacity to destabilize bacterial cell envelopes, chelate necessary metal ions, and enhance membrane permeability, all of which promote increased phytochemical penetration and aid in overcoming resistance mechanisms such as biofilm protection and decreased drug uptake. Because biofilms play a significant role in antibiotic resistance, this cooperation is essential. Therefore, by weakening bacterial defenses and enhancing the efficacy of plant-derived antimicrobial chemicals, the study promotes EDTA-phytochemical formulations as an environmentally friendly and practical approach to combating antimicrobial resistance (Vale et al., 2019).

The food industry often suffers from microbial infection and uses various antimicrobial agents. Phytotherapeutic agents remain a safe choice for preventing microbial infections in the packaged food industry. Essential oils such as thymol, carvacrol, and eugenol in combination with EDTA and HLE (3–6% H_2_O_2_; 2.2–4.4% lactic acid and 12.5–25 mM EDTA in water) proved to be highly effective against 30 strains of *Enterococcus* sp., *Staphylococcus* sp. and *Pseudomonas* sp. found in Goat and Lamb meat (Caballero Gómez et al., 2022). Bacteria are less prone to acquire resistance since both EDTA and essential oil components work by physically and biochemically disrupting specific molecular targets. They are appropriate for surface disinfection, environmental sanitation, and food chain applications due to their natural origin and safety profiles.

### 5.4 Application of EDTA in the field of medicine

In dentistry and veterinary medicine, the use of EDTA has been proven effective for treating biofilm-associated conditions. Several commercial formulations are available for wound dressings, which typically manage infections (via matrix metalloproteinases, MMP) (Finnegan and Percival, 2015). Multiple EDTA combinations are also used in practice to minimize biofilm formation on intravascular and urinary catheters, thereby reducing infection associated with these devices. It has also been applied to control pathogens including biofilms, often in combination with other materials (antiseptics and antibiotics), alcohol, citric acid, polyhexamethylene biguanide (PHMB) quaternary ammonium compounds, silver, iodine, etc (Lambert et al., 2004; Percival and Kite, 2007; Thomsen et al., 2011; Finnegan and Percival, 2015). EDTA has anticoagulant, antimicrobial, and antibiofilm activity and helps inhibit biofilms on a wide range of medical devices used in human care, including contact lenses, scleral buckles, suture materials, intraocular lenses, catheters and their insertion points, endotracheal tubes, etc. It can also serve as a low-cost alternative to heparin for maintaining intravenous catheters (Percival and Kite, 2007; Finnegan and Percival, 2015).

The outer membrane in Gram-negative bacteria becomes stabilized in the presence of bivalent metal ions, such as Ca^2+^ and Mg^2+^. These ions effectively neutralize the negative charge of the oligosaccharide chain of lipopolysaccharide (LPS) components present on the bacterial membrane. Upon the addition of EDTA, these metal ions are removed from LPS, and the outer membrane of Gram-negative bacteria is subsequently severely damaged (Finnegan and Percival, 2015). This exposes the phospholipid components of the membrane, where the antibacterial drug exerts its effect (Leive, 1965; Finnegan and Percival, 2015). As a result, many Gram-negative bacteria, which are otherwise resistant to lysozyme, are lysed upon the administration of EDTA and Tris buffer (2-amino-2-hydroxymethylpropane-1,3-diol) (Goldschmidt and Wyss, 1967).

RescuDerm, a commercially available wound care product, contains 0.1% EDTA and Biostepand is available as a water-soluble gel (Finnegan and Percival, 2015) A combination of 0.02% chlorhexidine digluconate (CHX) and 0.1% Na_2_EDTA compounded in poloxamer 407 saline solution is effective against *Acanthamoeba keratitis*, a rare but serious parasitic infection of the eye, mostly seen among contact lens wearers (Cucina et al., 2019). *In vitro* efficiency of the CHX-Na_2_EDTA ocular gel has been established through drastic reduction of the trophozoite and cyst survival (to 25% and 2%, respectively). Besides, Ollivier et al (Ollivier et al., 2003). have documented a 99.4% reduction of matrix metalloproteinase (MMP)-2 and -9 activity in the tear film of horses with active corneal ulcers when treated with EDTA. Other conventional treatment options are comparatively less effective (doxycycline: 96.3%, NAC: 98.8%, by NAC, ilomostat: 98.9%, 0.1% alpha1-PI: 52.4%, and 0.5% alpha1-PI: 93.6%).

### 5.5 Applications of EDTA in endodontic treatment

Endodontic treatment is a common issue that requires a cleaning solution, preferably one with broad-spectrum activity. During root canal treatment, a smeared layer, mainly of inorganic matter, is formed on the walls of the canals, which can completely obstruct the dentinal. This layer can also contain bacteria, which may prevent the penetration of antibacterial solutions into infected dentinal tubules, a factor that should be managed during endodontic treatments (Baker et al., 1975; McComb and Smith, 1975; Byström and Sunvqvist, 1985). The smeared layer can be easily removed, for example, irrigating the root canals with 10 mL of 17% EDTA, followed by 10 mL of 5% NaOCl, which effectively removes both the organic and inorganic components of the smear layer (Byström and Sunvqvist, 1985).

### 5.6 Applications of EDTA in chelation therapy

Chelating agents are mineral- or metal-binding substances that hold metal ions like a pincer. Using a claw-like configuration, EDTA can chelate heavy metals and other metal ions into a redox-inactive state, which can then be excreted by the kidneys or liver (Sultan et al., 2017). It can be used for chelation tests, e.g., detecting the presence of toxic substances in urine samples from patients. In current clinical practice, sodium edetate and calcium disodium edetate are used for this purpose (Ferrero, 2016; Feng et al., 2019).

**Figure 6:**
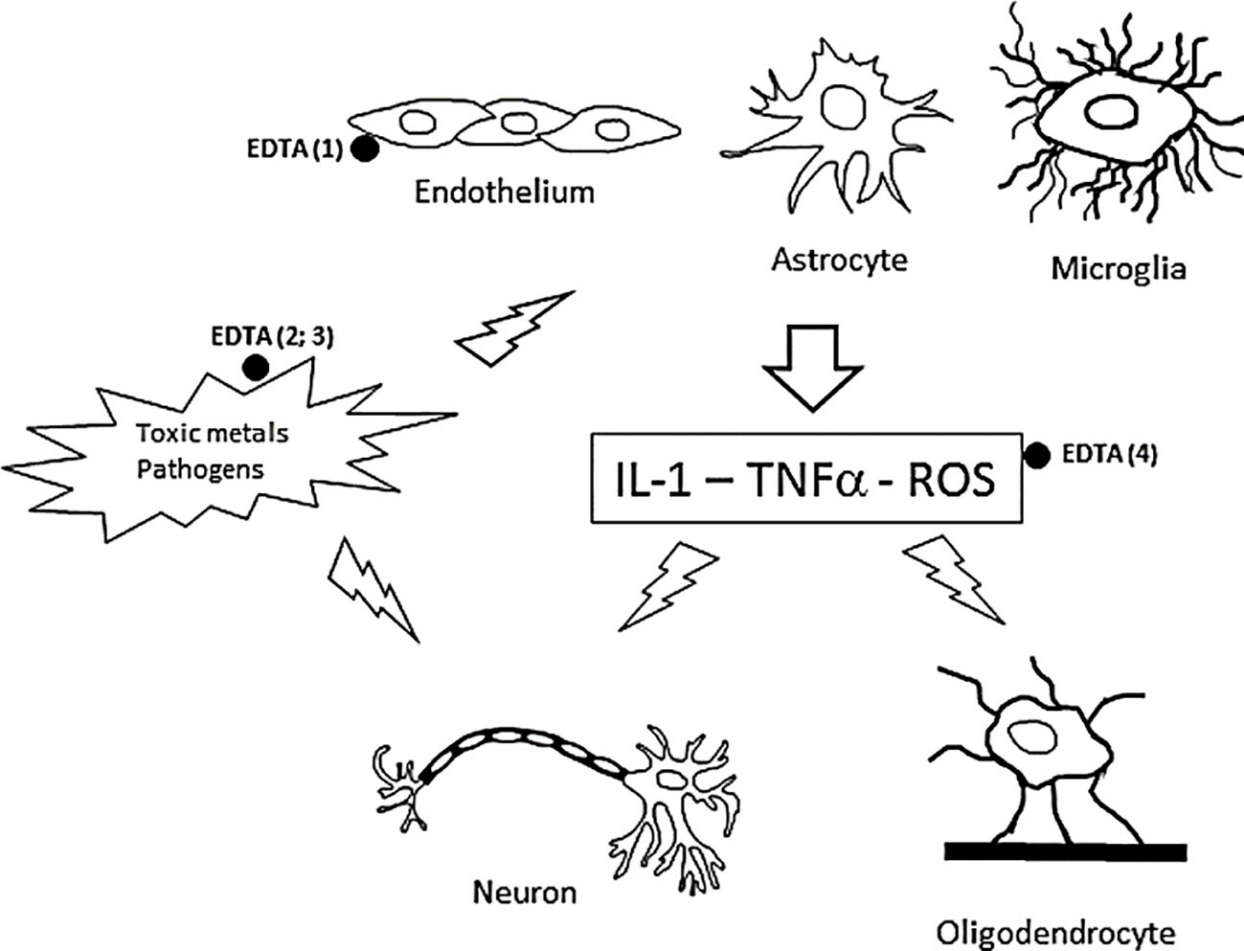
Proposed mechanism for the working of EDTA chelation therapy for neurotoxicity [adopted from reference (Alessandro and Maria Elena, 2019)].

EDTA is used in chelation therapy to treat neurotoxicity, which can be caused directly by many agents such as toxic metals, organophosphorus pesticides, air pollutants, neurotoxins, chemotherapeutic and anaesthetic drugs, etc. Around twenty-one metals are considered toxic, of which lead, cadmium, mercury, nickel, and aluminum induce neurotoxicity. EDTA binds to such toxic compounds and forms a stable complex that can be smoothly excreted (Corsello et al., 2009; Ferrero, 2016; Alessandro and Maria Elena, 2019; Fulgenzi et al., 2020). EDTA treatment is also beneficial to reduce the common symptoms in patients suffering from aluminum neurotoxicity (Alessandro and Maria Elena, 2019). The proposed mechanism of working of EDTA chelation therapy is presented in **Figure 6**.

Neurodegenerative diseases (ND) are associated with several toxic metals. Chelation therapy using EDTA eliminates toxic metals and improves patient symptoms (Fulgenzi et al., 2020). For example, TNFalpha-induced endothelial cell damage may be reduced (Sabolić, 2006), and low levels of free glutathione (GSH) in erythrocytes may be increased (Dellanoce et al., 2019). Consequently, the levels of LDL and ROS in blood samples are reduced. Thus, EDTA acts as an antioxidant compound, increasing the total antioxidant capacity (Foglieni et al., 2006; Fulgenzi et al., 2014).

A few authors also proposed the safe use of EDTA in cardiovascular diseases (Ouyang et al., 2015; Ferrero, 2016). Since EDTA chelation can help in treating toxicities involving both transition and toxic metals, its use can be beneficial for the treatment of cardiovascular diseases. Moreover, several scientists have proposed various mechanisms, two of which are the “Roto-Rooter hypothesis” and the “plaque decalcification theory”, which advocate for the mobilization of calcium from atherosclerotic plaques, causing their disintegration and subsequent softening of the arteries (Clarke et al., 1956; Chappell and Janson, 1996; Elihu et al., 1998; Sultan et al., 2017; Ravalli et al., 2022).

### 5.7 EDTA as an anticoagulant

An anticoagulant is a chemical substance used to inhibit blood clotting in both *in vitro* and *in vivo* conditions. The blood collection tubes in *in vivo* diagnostics usually contain EDTA, which maintains the fluidity of blood for hematological tests or allows the collection of plasma for chemical and clinical analyses (Banfi et al., 2007). Several enzymatic reactions of the coagulation pathway need calcium, without which blood clotting is irreversibly prevented within the collection tube. EDTA has the potential to form complexes or to chelate metal ions. The interaction between calcium and the carboxyl groups of EDTA helps prevent clotting and stabilize the blood in a fluid state.

Kennedy et al. emphasized the precise identification of samples exposed to different anticoagulants to perform accurate and precise clinical metabolomic assays (Kennedy et al., 2021). However, various additives used during blood collection and storage can affect the levels of several clinically important biochemicals, and therefore, they should be selected carefully.

EDTA is the anticoagulant of choice for hematological testing, as it best preserves cellular components and blood cell morphology. Out of the three EDTA formulations (Na_2_EDTA, K_2_EDTA, and K_3_EDTA) traditionally used as anticoagulants, the choice largely depends on the nature of the analyses to be undertaken. K_2_EDTA, available in spray-dried form, does not introduce a dilutional effect in small sample volumes and induces a comparatively less pronounced osmotic effect on blood cells than other agents. Additionally, it provides optimal staining with the May-Grunwald Giemsa stain; therefore, it is preferred for making smears of peripheral venous blood. Hence, the International Council for Standardization in Haematology (ICSH) currently recommends it as an anticoagulant for hematological testing. However, K_3_EDTA is more generally used in the US and the UK. Since EDTA is used as a sodium or potassium salt, it is not suitable to measure these ions (Reardon et al., 1991).

The consistency of hematological parameters in EDTA-stored blood is very high. For example, hemoglobin is stable for 48 hours, red blood cells for 24 hours, and white blood cells for 24 hours when stored in EDTA at 48 °C. The differential cell count also remains steady in refrigerated samples. In whole-blood samples, reticulocytes tend to mature and transform into RBCs, but stability is high (72 hours) when stored at 48 °C with EDTA as an anticoagulant (Felding et al., 1981; Banfi et al., 2007).

### 5.8 Protective effect of EDTA preadministration on renal ischemia

Ischemia is a condition in which blood flow to tissues is restricted, resulting in a shortage of oxygen at the tissue level. Ischemia-reperfusion injury (IRI) is the tissue damage caused by the restoration of blood supply after a period of ischemic lack of oxygen. Tissue damage and injuries may occur after infarction, sepsis, and organ transplantation, which initiates an inflammatory cascade including ROS production and activation of cytokines, chemokines, and leukocytes (Wu et al., 2018). IRI contributes to pathological alterations of the kidney called acute kidney injury (AKI). Kidneys with fatal clinical syndromes like rapid kidney dysfunction are characterized by early alloantigen-independent inflammation (Jang and Rabb, 2009; Sharfuddin and Molitoris, 2011; Malek and Nematbakhsh, 2015). Chelation therapy involving sodium slows the progression of renal insufficiency, improves renal function, and delays the progression of chronic kidney disease in patients subjected to lead intoxication. EDTA administration preserved kidney function and prevented necrotic lesions and structural alterations (Lin et al., 1999; Foglieni et al., 2006; Ferrero, 2016).

The exact mechanism by which EDTA-induced lead chelation delays renal impairment is not known, but it is hypothesized that chronic low-level lead exposure may increase ROS levels, which inactivate nitric oxide (NO). EDTA-chelation therapy likely reduces ROS production, thereby enhancing the availability of vascular NO by preventing ROS from inactivating it, thereby potentially improving renal function (Ding et al., 1998; Lin et al., 2003; Foglieni et al., 2006).

### 5.9 Role of EDTA in aggravating colitis and colon carcinogenesis

Inflammatory bowel disease (IBD), triggered both by genetic and environmental factors including diet, includes a group of conditions and is a ‘prototype disease’ for chronic auto-inflammatory disorders. Processed foods are thought to be the main reason for the frequent occurrence of IBD, particularly Crohn’s disease. EDTA compounds, such as Na-EDTA and Ca-EDTA, are widely used in the food industry as sequestrants and stabilizing agents to improve color and flavor stability, as well as for iron fortification (FeNaEDTA·3H_2_O or Fe-EDTA). Data on the impact of EDTA on the aggravation of IBD, infectious diarrhoea, or colorectal cancer is scarce (Bartel et al., 2008; Rogler et al., 2016; Evstatiev et al., 2021).

**Figure 7:**
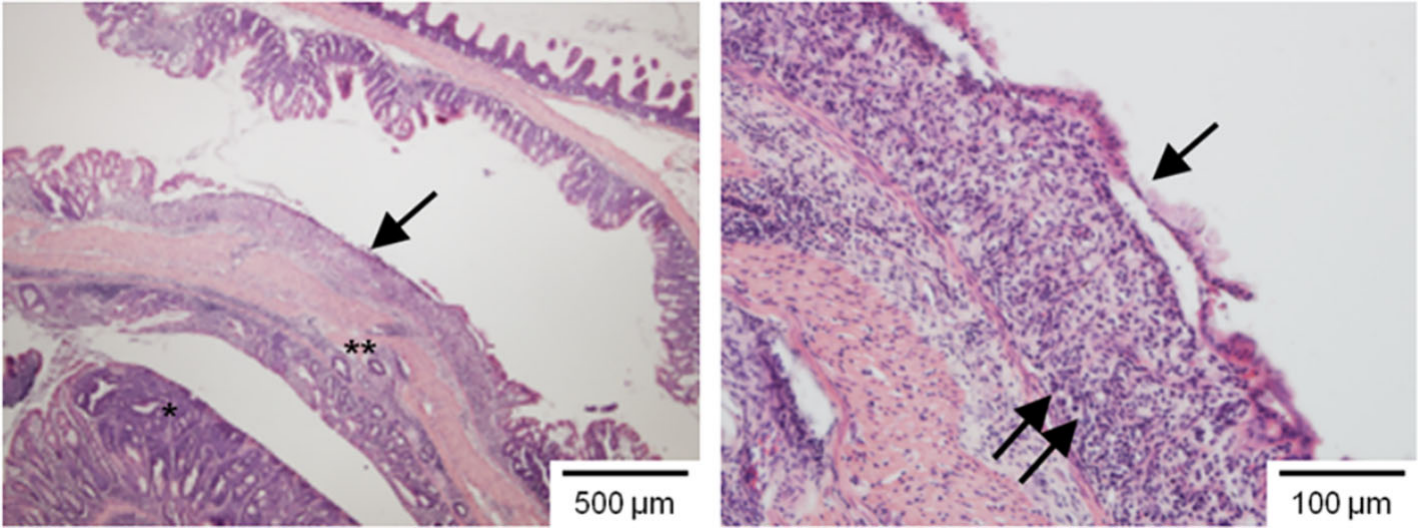
Impact of Fe-EDTA leading to massive inflammation (double arrow) with complete crypt destruction. An invasive tumour (*) is seen on the lower magnification image (left) (Adopted from reference (Evstatiev et al., 2021)).

Evstatiev et al. (2021) used mouse models and demonstrated that even lower doses of EDTA can exacerbate pre-existing intestinal inflammation and may induce colorectal carcinogenesis (**Figure 7**). It disrupts several components of the intestinal barrier, including intercellular contacts, and thereby increases intestinal permeability. The adherens junctions (AJs) and cellular contacts become weakened through the increased cytoplasmic localization of E-cadherins due to the loss and reduced membranous expression of β-catenin in the distal colon, even in healthy individuals, with a significant aggravation in the presence of inflammation. Increased incidence of intestinal inflammation and colitis-associated carcinogenesis in various Fe-EDTA-treated animal models, but its absence in models treated with other iron compounds indicates the specificity of intestinal toxicity to Fe-EDTA. Studies have confirmed that colitis-associated carcinogenesis is due to EDTA itself rather than to any of its salts.

### 5.10 Toxicity on dentin structures

The application of EDTA in endodontic treatments to remove the smear layer is a common practice. As EDTA solution has strong demineralizing properties, its application softens the dentin layer, denatures collagen fibers, and enlarges dentinal tubules (Byström and Sunvqvist, 1985; Calt and Serper, 2002). Orhan et al. observed that 17% EDTA showed the highest percentage of open dentin tubules after smear layer removal, compared with 5.25% sodium hypochlorite and sterile distilled water (Orhan et al., 2021). Other chelating agents, such as 9% etidronic acid and 2% peracetic acid, either alone or in combination with sodium hypochlorite, induced structural alterations in root canal dentine (Ulusoy et al., 2020). Thus, adapting root canal filling materials to the root canal wall presents a challenge. Atalay and Erisen examined the effect of five chelating agents (EDTA, NaOCl, phytic acid, citric acid and distilled water) on the roughness of root dentin. The citric acid and phytic acid solutions decreased the calcium-to-phosphorus ratio in dentin tissue; however, EDTA, phytic acid, and citric acid solutions increased dentin roughness. While increased roughness supports the attachment of root canal filling materials to the dentin surface, decreased Ca/P ratio should be considered during irrigation (Atalay and Erişen, 2023).

The smear layer gets completely removed on irrigation with 17% EDTA for 10 minutes, followed by 5% NaOCl. However, this treatment also results in massive erosion of peritubular and intertubular dentin, further widening tubular diameters and converging tubular apertures. The intertubular dentin completely disappears among the tubules due to excessive erosion in some areas, bringing the individual tubule openings into proximity. Tubular openings increase nearly two-fold (up to 4 µm in some cases) after a 10-minute treatment compared to a 1-minute treatment (Calt and Serper, 2002). The effect was probably seen due to the alternating action of EDTA (demineralizes the inorganic component) and NaOCl (dissolves the organic component of the dentin) (Xu et al., 2022). Therefore, applying the EDTA solution for less than one minute helps prevent dentin erosion (Craigbaumgartner and Mader, 1987; Calt and Serper, 2002). The sequential application of bulk-fill resin composite to dentin enhances its adhesive strength when preconditioned with 18% EDTA followed by 35% H_3_PO_4_, compared with either preconditioner alone (Alcántara-Obispo et al., 2022).

The extrusion of irrigants beyond the apical constriction during EDTA-mediated root canal treatment results in undesirable direct contact between the irrigating substances and periapical tissues (Malheiros et al., 2005; Tinaz et al., 2005; Amaral et al., 2007). Periapical tissue-compatible decalcifying agents should be used to avoid any toxicity-induced irritation, inflammation, allergy, genotoxicity, or carcinogenic action (Sun et al., 1997; Zaccaro Scelza et al., 2010). Rath and coworkers reported that sequential chelation with NaOCl/EDTA exposed bare collagen fibers, whereas continuous chelation with NaOCl/etidronate produced a fragile surface of the collagen layer (Rath et al., 2020). Such surface and sub-surface alterations may contribute to structural failures of dentin and/or to failures at the dentin-biomaterial interfacial.

### 5.11 Ecotoxicological role of EDTA

EDTA is a persistent environmental contaminant with both direct and indirect effects. It may cause the dissolution of heavy metals adsorbed in sediments or prevent their precipitation in solution, resulting in enhanced mobility and bioavailability through complex formation. Although isolated molecules of EDTA do not pose a risk of bioaccumulation, their ligand-metal complexes can significantly enhance the bioavailability of hazardous heavy metals (Vassil et al., 1998). In addition, the nitrogen component (approximately 10%) of EDTA may enhance the eutrophication of water bodies and could eventually become available to aquatic microorganisms (Means et al., 1978; Friedly et al., 2002; Oviedo and Rodríguez, 2003). EDTA lowers the inhibition exerted by toxic contaminants on biological nutrient removal bacteria. However, it impairs the nitrification process (Wang et al., 2016; Mpongwana et al., 2022). Further, the environmental mobility of radioactive substances may increase through their solubilization.

EDTA disrupts soil microfauna, with soil fungi being the most affected group (Grčman et al., 2001). Being harmful to Gram-negative bacteria, it exhibits resistance to bacterial biodegradation (Madsen and Alexander, 1985; Bolton et al., 1993; Allard et al., 1996). During wastewater/sewage treatment, it passes unmodified through both biological and chemical processes due to its resistance to biodegradation and limited absorbability. Hence, such treatments are ineffective, and EDTA is retained in water bodies (Oviedo and Rodríguez, 2003). EDTA is a conventional chelator effective in the remediation of sewage sludge contaminated with heavy metals; however, its low biodegradability is a significant drawback. Hu et al. (2022) extracted heavy metals from sewage sludge using methylglycine diacetic acid (MGDA). They demonstrated it to be a potential, environmentally friendly alternative to chelators, such as EDTA. Additionally, combined electrochemical-EDTA treatment of waste-activated sludge (WAS) significantly enhances phosphorus release from both WAS and extracellular polymeric substances (Xu et al., 2021).

EDTA, through its toxicity, inhibits cell multiplication, orophyll synthesis, as wand l biomass production in photosynthetic organisms. However, similar concentrations of micronutrient-chelated EDTA did not show alterations. Tubbing et al. (1994) studied the river microalgae and found that EDTA-complexed heavy metals [chelation with 5 – 10 µM copper (II)] were biologically available and toxic. Khan et al. (2019) have shown that co-application of EDTA (2.5 mM) enhanced the uptake of heavy metals by *P. hybrida* L., leading to accompanying changes in biochemical stress indicators (considerably higher H_2_O_2_ contents, MDA, and electrolyte leakage with reduced chlorophyll a, chlorophyll b, total chlorophyll, and carotenoid content). Studies by Yadav and Sharma (2018) on *Hordeum vulgare* L. (barley) seedlings demonstrated that EDTA is antagonistic to Cr(VI)- induced biochemical toxicity, decreases lipid peroxidation, enhances the antioxidative defense system, improves chlorophyll content, and reduces chromium uptake.

### 5.12 Effect of EDTA-Fe(II) on lipid peroxidation

Lipid peroxidation is a form of oxidative damage that affects cellular membranes, CNS, lipoproteins, and other lipid-containing molecules. It is one of the important possible sources of free radical-mediated injury. During the process, oxidants (e.g., free radicals) target lipids having carbon-carbon double bonds, especially the polyunsaturated fatty acids, forming highly reactive electrophilic aldehydes. Ayala et al. (2014); Sultana et al. (2013) have reported impairments in a series of membrane-related activities, including increased membrane rigidity, dysfunction of membrane receptors, reduced activity of membrane-bound enzymes, and altered membrane permeability. Furthermore, aldehydes selectively and covalently bind to specific amino acid residues, resulting in post-translational protein modifications. These protein adducts disrupted cellular homeostasis and therefore needed to be removed.

Peroxidation of polyunsaturated fatty acids mostly occurs in microsomes and needs NADPH, but not NADH, as the reducing agent and iron salts. EDTA-Fe(II) complex stimulates the process in a NADH-dependent enzymatic process in brain microsomes, but the addition of EDTA-Fe(III) completely blocks the process. Thus, only EDTA-Fe(II) induces lipid peroxidation (Marton et al., 1987; Patole and Ramasarma, 1988). Both Ca and EDTA significantly affect the arsenic accumulation and toxicity of *Vicia faba* (Rafiq et al., 2018). As-induced lipid peroxidation and reactive oxygen species production are decrease with the application of both amendments.

### 5.13 Cytotoxicity and genotoxicity

Cytotoxicity is the ability of a substance to be toxic to cells. The cell protein content is reduced by up to 50% when treated with tetrasodium EDTA (Dierickx, 1989; Matthews et al., 1993). Matthews et al. found the trisodium EDTA to be moderately cytotoxic in BALB/c-3T3 cells (LD_50_ = 1.98 mmol, coculture survival assay). Treatment with Cu-EDTA and tetrasodium EDTA can cause cell necrosis and reduce the colony-forming ability of normal rat kidney cells in culture. EDTA reduces cell viability over time by decreasing cell density (Hugenschmidt et al., 1993; Lanigan and Yamarik, 2002). The viability of EDTA-treated macrophages is reduced by 50–70% in 24 hours (Herrmann et al., 2012). The chelator ions, such as Ca^2+^ and Mg^2+^, promote alterations in the cell membranes of macrophages, thereby accelerating the apoptotic process, as they act as cofactors in many enzymatic reactions. EDTA can also have an indirect effect on cell metabolism as it can decrease the pH, which in turn, reduces the availability of nutrients to cells, causing a significant reduction in macrophage viability (Amaral et al., 2007). Giardino et al (Giardino et al., 2020), assessed the cytotoxic and genotoxic effects of EDTA and maleic acid either alone or in combination with cetrimide on Chinese hamster cells V79. 17% EDTA was more cytotoxic compared to 7% maleic acid. However, the combined effects of cetrimide with EDTA or maleic acid effectively removed the hard-tissue debris accumulated in the canal walls and also recorded enhanced antimicrobial activity.

EDTA, although considered a weak mutagen in microbial systems, still affects the cell division processes. EDTA and disodium-EDTA induce chromosomal breakage through the chelation of either calcium or magnesium. Further, it can break and deform salivary chromosomes of *Drosophila melanogaster* (Palladino et al., 2020) and plants. An increased incidence of chromosomal aberrations in seeds or plant root tip meristems was observed following treatment with EDTA prior to X-ray and chemical mutagen exposure (Saha and Chakrabarty, 1973; Heindorff et al., 1983; Lanigan and Yamarik, 2002).

EDTA can inhibit DNA synthesis as seen in rabbit adrenal cortex primary cell cultures and also inhibit the repair of irradiation- or mutagen (chemical)–induced DNA lesions (Rubin, 1972). It could be due to the direct impairment of repair enzymes or alteration of chromatin structure, which denies access to the repairing enzymes at lesions (Lieberman and Ove, 1962; Heindorff et al., 1983; Lanigan and Yamarik, 2002). Calcium-EDTA is teratogenic as seen in rats at even non-maternotoxic doses and is shown to cause cleft lip, cleft palate, brain malformations, micro- or anophthalmia, micro- or agnathia, clubbed legs, fused or missing digits, or tail malformations (curly, short, or missing tail) (Swenerton and Hurley, 1971; Lanigan and Yamarik, 2002). The frequency of malformations and retention time of chemical teratogens, when co-administered with EDTA, is increased (Wilk et al., 1978; Lanigan and Yamarik, 2002). **Table 1** describe the comparative genotoxicity among different EDTA salts.

**Table 1:** Genotoxicity of EDTA and its salts.

EDTA/ salts	Treatment host/system	Type of genotoxicity	Observation	References
EDTA	BM, EC	GM	Increased phage induction without any effects on streptomycin resistance	(Northrop, 1968)
	DM	GM	Increased mutation	(Baranauskajte et al., 1972)
	MLC	GM	No increase up to 25.2 mmole/L while increase in mutation at 30.2 mmole/L	(Wangenheim and Bolcsfoldi, 1988)
	MLC	DD	No increase up to 30.4 mmole/L while increase in DNA break, 40.5-50.6 mmole/L	(Garberg et al., 1988)
	MBMC	CA	Increased aberrations	(Manna and Das, 1971)
	MPC	CA	Increased aberrations	(Das and Manna, 1972)
	HL	CA	Increased aberrations	(Basrur and Baker, 1963)
	DM larvae	CO	Crossover increases	(Levine, 1955)
	Green algae	CO	Crossover increases	(Eversole and Tatum, 1956)
Na_2_EDTA	Swiss albino mice (male); bone marrow, micronucleus assay	GM	Increased micronuclei	(Narasimhamurthy M. K. (1991))
	Male grasshopper	CA	Slightly increase in aberrations	(Saha and Chakrabarty, 1973)
	Mosquito	CA	increase in aberrations	(Chaudhry et al., 1988)
K_3_EDTA	HL	SCE	Increase in sister chromatid exchange	(Tucker and Christensen, 1987)

*Bm, Bacillus megatherium; Ec-Escherichia coli;* DM, *Drosophila melanogaster;* HL, Human leucocytes; MLC, Mouse lymphoma cells; MBMC, Mouse bone marrow cells; MPC, Mouse spleen cells; CA, Chromosomal aberrations; CO, Crossover; GM, Gene mutations; SCE, Sister chromatid exchange.

The toxicity of EDTA is attributed to its chelation potential that alters the distribution of ions in tissues. Administration of EDTA with bean cooking media had significantly increased alanine aminotransferase and creatinine levels, thus impairing liver and kidney functions in mouse models (El-Naggar et al., 2020). EDTA can induce biochemical alterations, such as an increase in transaminases (ALT and AST) and blood urea levels. It is toxic to the liver and kidney of mice even at low doses. It can cause disorganization of hepatic architecture, cellular infiltration, cytoplasmic degeneration and vacuolation, formation of necrotic areas, congestion of blood vessels, and the presence of apoptotic bodies in the liver, as well as disorganization of glomeruli and erosion of the walls of Bowman’s capsules in the kidney (El-Naggar et al., 2020).

### 5.14 Ecological and biological concerns

EDTA is persistent in both terrestrial and aquatic environments due to its excellent chemical stability and resistance to biodegradation. Because of EDTA’s high chelating activity, which can mobilize heavy metals from sediments and soils, increasing their bioavailability and potential toxicity, its persistence poses ecological concerns. Such mobilization may change the architecture of microbial communities, upset ecological equilibrium, and make it easier for plants and aquatic species to absorb heavy metals. As a result, EDTA’s extensive use in industry, medicine, and food preservation may have unforeseen biological effects (Industrial & Commercial Uses of EDTA: Applications in Food and Cosmetics). Future studies should focus on developing biodegradable EDTA analogs and on regulated disposal methods that reduce environmental buildup and biological disturbance to mitigate these hazards.

### 5.15 Toxicological thresholds and therapeutic window limitations

The environmental persistence of EDTA raises concerns about accumulation and long-term ecological damage, despite its widespread use and generally being regarded as safe at controlled doses. Its cost-effectiveness makes it appealing for industrial and medicinal uses, but safe handling and disposal might be especially difficult in healthcare systems with limited resources (Nowack, 2002). Higher EDTA concentrations have been shown to cause gastrointestinal problems, nephrotoxicity, and hypocalcemia (George and Brady, 2023). Crucially, the therapeutic window is limited, as antibacterial and antibiofilm activity often require doses close to hazardous thresholds, raising concerns about clinical viability. Therefore, despite its wide range of applications as a chelating agent and antibacterial adjuvant, a few drawbacks call for careful consideration. The most important of them is its dose-dependent toxicity, which is still a major problem (Da Silva et al., 2025). EDTA can have negative physiological consequences at doses high enough to break up biofilms and increase antibacterial activity. This emphasizes the need for methodical dose-response studies to maximize effectiveness while guaranteeing patient safety, especially when EDTA is used in combination treatments.

### 5.16 From *in vitro* promise to clinical uncertainty

Even though EDTA has shown significant antibacterial and antibiofilm activity in preclinical models and *in vitro*, there is still no evidence of its use in routine clinical practice. When EDTA is combined with agents like colistin or thymol, experimental studies often report strong synergistic effects; however, these results are not consistently replicated *in vivo*, where host physiology, pharmacokinetics, and tissue penetration significantly influence therapeutic efficacy. Few solid clinical studies have been conducted to support EDTA’s use as an adjuvant in antimicrobial treatment, and most of the available information comes from laboratory models. The lack of well-designed randomized clinical trials limits the ability to determine its safety, optimal dosage, and therapeutic value in dentistry, catheter care, wound management, and the diagnosis of parasitic diseases, despite encouraging preclinical results. Therefore, unless it is confirmed by thorough clinical testing, EDTA’s potential as a therapeutically significant antibacterial adjuvant is mostly theoretical.

## 6 Acceptable daily intake and permissible concentrations

The permissible limits of EDTA vary across segments. The recommended acceptable daily intake (ADI) limit in the food industries for humans is 1.9 mg EDTA/kg body weight (Wreesmann, 2014) The maximum levels of EDTA used in canned shrimps and prawns are 250mg/kg, in canned mushrooms is 200mg/kg, in frozen French fries is 100 mg/kg and for drinking water is around 600 µg/L assuming a 2 L water intake/day in an average adult (60kg) (Evstatiev et al., 2021). The concentration of K_2_EDTA, used as an anticoagulant in hematological testing and recommended by the NCCLS, is 1.5–2.2 g/L (Banfi et al., 2007). For the chelation test, 2g EDTA is diluted in 500 mL of physiological saline (NaCl 0.9%) and administered over a period of 2 hours. For root canal treatment, 10 ml of 17% EDTA solution is used. However, in the absence of a prescribed time limit, around 10 minutes is typically followed most of the time. **Table 2** presents the concentration range of various EDTA salts used in cosmetics. The LD_50_ values (mg/kg) for various model organisms are 2000–2200 of Na_2_EDTA and 10,000 ± 740 of CaNa_2_EDTA for rats, 2300 of Na_2_EDTA and 7000 of CaNa_2_EDTA for rabbits, 500 of CaNa_2_EDTA and for dogs (Oser et al., 1963).

**Table 2:** Concentration range for EDTA and salts used in cosmetic formulations (Adopted from Lanigan and Yamarik, 2002; Wilma et al., 2019.

EDTA/salts	Product category	Concentration range allowed
EDTA	Bubble baths	0.025%
	Eye makeup preparation (lotions, remover)	0.03-0.05%
	Hair conditioners	0.0005-0.13%
	Shampoos (color v noncolor)	0.0016-0.04% and 0.11%
	Hair tints	0.2%
	Skin cleansing preparations (cold creams, liquids, pads)	0.01-0.048%
Disodium EDTA	Bubble baths	0.05%
	Eye makeup preparation (eyebrow, liner) and lotion	0.05-0.3% and 0.05-0.19%
	Hair conditioners	0.0001-0.2%
	Shampoos (color v noncolor)	0.1-0.2% and 0.00002-0.69%
	Hair dyes	0.025-1.5%
	Skin cleansing preparations (cold creams, liquids, pads)	0.1-0.3%
	Perfumes	0.0011-0.12%
	Lipstick	0.0003-0.1%
	Vaginal douches	0.09%
	After shave lotions	0.05-0.2%
	Shaving cream	0.02-0.1%
Ca-disodium EDTA	Deodorants	0.025%
Dipotassium EDTA	Skin cleansing preparations (cold creams, liquids, pads)	0.054%
Tetrasodium EDTA	Bath soap, detergent	0.024-0.56%
	Eye makeup preparation (eyeliner and lotion)	0.004-0.054% and 0.043%
	Hair conditioners	0.00002-0.75%
	Shampoos (color); hair tints	0.078-1.9% and 0.092-0.25%
	Hair dyes	0.029-1.9%
	Skin cleansing preparations (cold creams, liquids, pads)	0.01-0.16%
	Perfumes	0.085%
	Lipstick	0.08%
	Face powder	0.048%
	After shave lotions	0.068-0.085%
	Shaving cream, soap	0.098%
	Deodorants	0.016-0.5%
Trisodium EDTA	Bath soap, detergent	0.084%
	Eye makeup preparation (eyeliner and lotion)	0.059% and 0.05%
	Eye makeup remover	0.15%
	Shampoo (noncolor)	%
	Hair dyes	0.35%
	Skin cleansing preparations (cold creams, liquids, pads)	0.1-0.12%
	Perfumes	0.085%
	Lipstick	0.2%
Trisodium HEDTA	Bubble baths	0.000017%
	Hair growing a stonic	0.004%
	Hair conditioners	0.066%
	Shampoos (noncolor)	0.02-0.13%
	Hair dyes	0.3%
	Skin cleansing preparations (cold creams, liquids, pads)	0.06%

## 7 Future prospects

Future investigations should prioritize expanding EDTA’s application by testing it in combination with a broader spectrum of natural compounds and innovative delivery systems. Such work could uncover novel synergistic formulations that enhance antimicrobial potency and extend therapeutic options for resistant infections. Additionally, exploring EDTA’s role in industrial domains such as food preservation and biomedical device coatings may open new avenues for practical implementation. Nevertheless, several limitations must be addressed before widespread adoption. EDTA exhibits poor gastrointestinal absorption and rapid elimination, which restrict systemic efficacy. Concerns regarding dose-dependent toxicity and ecological persistence also demand careful evaluation. Sustainable integration of EDTA into clinical and industrial practice will therefore require optimized dosing strategies, improved formulations, and thorough environmental risk assessments.

## 8 Conclusion

EDTA demonstrates remarkable potential as a multifunctional adjuvant in antimicrobial therapy. By chelating divalent cations, it destabilizes biofilm architecture, weakens microbial membranes, and enhances drug penetration, thereby restoring the efficacy of conventional antibiotics and natural compounds against resistant pathogens. Its synergistic interactions with phytochemicals, peptides, and essential oils further broaden therapeutic options, reducing required dosages and improving outcomes in both planktonic and biofilm-associated infections. Beyond clinical relevance, EDTA’s antimicrobial properties also hold promise for industrial applications in food safety and biomedical device management. Importantly, realizing these benefits will depend on carefully addressing concerns of dose-related toxicity and ecological persistence, ensuring that EDTA’s integration into antimicrobial stewardship strategies is both safe and sustainable.
